# Primate piRNA Cluster Evolution Suggests Limited Relevance of Pseudogenes in piRNA-Mediated Gene Regulation

**DOI:** 10.1093/gbe/evz060

**Published:** 2019-03-19

**Authors:** Daniel Gebert, Hans Zischler, David Rosenkranz

**Affiliations:** Institute of Organismic and Molecular Evolution, Anthropology, Johannes Gutenberg University, Mainz, Germany

**Keywords:** piwi, transposons, comparative genomics, parent genes, strepsirrhini

## Abstract

PIWI proteins and their guiding Piwi-interacting (pi-) RNAs direct the silencing of target nucleic acids in the animal germline and soma. Although in mammal testes fetal piRNAs are involved in extensive silencing of transposons, pachytene piRNAs have additionally been shown to act in post-transcriptional gene regulation. The bulk of pachytene piRNAs is produced from large genomic loci, named piRNA clusters. Recently, the presence of reversed pseudogenes within piRNA clusters prompted the idea that piRNAs derived from such sequences might direct regulation of their parent genes. Here, we examine primate piRNA clusters and integrated pseudogenes in a comparative approach to gain a deeper understanding about mammalian piRNA cluster evolution and the presumed gene-regulatory role of pseudogene-derived piRNAs. Initially, we provide a broad analysis of the evolutionary relationships of piRNA clusters and their differential activity among six primate species. Subsequently, we show that pseudogenes in reserve orientation relative to piRNA cluster transcription direction generally do not exhibit signs of selection pressure and cause weakly conserved targeting of homologous genes among species, suggesting a lack of functional constraints and thus only a minor significance for gene regulation in most cases. Finally, we report that piRNA-producing loci generally tend to be located in active genomic regions with elevated gene and pseudogene density. Thus, we conclude that the presence of most pseudogenes in piRNA clusters might be regarded as a byproduct of piRNA cluster generation, whereas this does not exclude that some pseudogenes nevertheless play critical roles in individual cases.

## Introduction

Piwi-interacting RNAs (piRNAs) represent a class of small (∼24 to 32 nt) noncoding RNAs (sRNAs) in animals that associate with Piwi-clade Argonaute proteins (**Washington, DC:**IWI) to regulate specific targets, such as transposons, on the transcriptional and post-transcriptional level (Gebert and Rosenkranz 2015; Iwasaki et al. 2015; Ozata et al. 2019). In the classical view, the biogenesis of piRNAs ensues within two pathways, resulting in primary and secondary piRNAs (Czech and Hannon 2016). Primary piRNAs are generated from larger single-stranded RNA molecules, like the transcripts of a set of large genomic loci, named piRNA clusters (Aravin 2006; Girard et al. 2006; Grivna et al. 2006; Watanabe et al. 2006). PIWI proteins loaded with primary piRNAs biased for a 5′ uracil (1U) can then enter the so-called ping-pong cycle that produces secondary piRNAs from reverse complementary target transcripts that are cleaved with a 10 nt offset from the 5′ end of the guide RNA and bound by another PIWI protein. The resulting secondary piRNA finally allows targeting of piRNA cluster transcripts, yielding piRNAs that resemble those that initiated the cycle (Brennecke et al. 2007; Gunawardane et al. 2007; Aravin et al. 2008). In this manner, the ping-pong cycle results in post-transcriptional target repression and a self-sustaining amplification of sense and antisense piRNAs. Furthermore, it has been shown that piRNAs arising from the ping-pong cycle can in turn trigger the production of phased or trailing piRNAs from longer piRNA precursor transcripts (Han et al. 2015; Homolka et al. 2015; Mohn et al. 2015). Based on these insights, a unified model of piRNA production that uses a more appropriate nomenclature referring to trailing piRNAs instead of primary piRNAs, and ping-pong piRNAs instead of secondary piRNAs has been proposed recently (Gainetdinov et al. 2018; Ozata et al. 2019).

In mammalian testis different populations of piRNAs are distinguished depending on developmental stage and phase of spermatogenesis. While fetal piRNAs are found in primordial germ cells of the developing embryo, postnatal piRNAs are divided into two sequentially expressed types, namely pre-pachytene piRNAs, present before meiosis, and pachytene piRNAs, which appear starting from the pachytene meiotic phase of spermatogenesis (Aravin, Sachidanandam, et al. 2007) and constitute more than 95% of all piRNAs in adult testis (Li et al. 2013). Furthermore, these distinct piRNA populations interact with different PIWI paralogs. Piwi-like 1 (Piwil1) is solely associated with pachytene piRNAs and Piwil4 is only present in perinatal testis, whereas Piwil2 binds piRNAs of all types (Aravin et al. 2008). Fetal piRNAs direct both post-transcriptional and transcriptional silencing of transposons during epigenetic reprograming, requiring extensive ping-pong cycle amplification (Aravin, Sachidanandam, et al. 2007; Aravin et al. 2008; De Fazio et al. 2011). Pachytene piRNAs, in contrast to fetal and pre-pachytene piRNAs, exhibit a reduced share of transposon-derived sequences and are mostly generated in primary biogenesis from large pachytene-specific piRNA clusters (Aravin, Sachidanandam, et al. 2007; Beyret et al. 2012). Nevertheless they are still required for post-transcriptional repression of transposons such as LINE-1 (Reuter et al. 2011). It was suggested that fetal/pre-pachytene and pachytene piRNA clusters, which overlap only to a minor degree (Aravin, Sachidanandam, et al. 2007; Li et al. 2013), act as transposon traps that passively acquire new transposon sequences by random insertion and subsequent evolutionary fixation, providing the source for piRNAs that can target homologous transposons (Malone and Hannon 2009).

In addition to transposon repression, pachytene piRNAs were also shown to play a role in gene regulation, involving ping-pong cycle processing (Gou et al. 2014; Gebert et al. 2015; Goh et al. 2015; Zhang et al. 2015). In this context pseudogene-containing piRNA clusters have been suggested to be an important source of gene-targeting antisense piRNAs (Hirano et al. 2014; Gebert et al. 2015; Pantano et al. 2015; Watanabe et al. 2015). Generally, whereas some piRNA-producing loci are active across many species (Chirn et al. 2015), piRNA clusters typically evolve rapidly on a large scale (Assis and Kondrashov 2009). This raises the question of whether pseudogene-containing piRNA clusters are maintained throughout evolution to retain their ability to target genes, which would indicate the biological relevance of pseudogene-derived PIWI-mediated gene regulation. In this work, we study the evolution of primate piRNA clusters and the conservation of therein contained pseudogenes and their capacity to target coding genes across species to elucidate putative gene-regulatory roles of pseudogene-derived piRNAs.

## Results and Discussion

### Basic Analyses of sRNA Data Sets

We based our study on adult testis-expressed small RNA transcriptome data from six primate species, including publicly available data sets of *Homo sapiens* (Hsap, human), *Macaca mulatta* (Mmul, rhesus macaque), *Macaca fascicularis* (Mfas, crab-eating macaque), and *Callithrix jacchus* (Cjac, common marmoset). Furthermore, by generating data sets for the strepsirrhine species, *Microcebus murinus* (Mmur, gray mouse lemur) and *Loris tardigradus* (Ltar, red slender loris), we considerably expand the spectrum of primates available for study, thus covering more than 60 million years of primate evolution (Pozzi et al. 2014). First, we performed basic analyses on sRNA data sets (supplementary fig. S1, Supplementary Material online). Unifying characteristics of piRNAs, such as a size range between 24 and 32 nt (supplementary fig. S1*A*, Supplementary Material online), 1U/10A biases (supplementary fig. S1*B*, Supplementary Material online) and ping-pong signatures, meaning a high relative amount of 10 nt 5′ overlaps, (supplementary fig. S1*C*, Supplementary Material online) were observed in each case. The shares of reads that have ping-pong partners is low, which is typical for pachytene piRNAs (Reuter et al. 2011), ranging from 5% to 12% of 24–32 nt nonidentical reads (supplementary fig. S1*C*, Supplementary Material online). Furthermore, one can infer from local peaks in the read length distributions that the PIWI paralogs Piwil2 and Piwil1 are present and likely associated with piRNAs of ∼26/27 and ∼29/30 nt, respectively (supplementary fig. S1*A*, Supplementary Material online), because it is known from mice and other mammals that different PIWI proteins bind piRNAs of distinct size ranges, particularly that Piwil1, Piwil2, and Piwil4 bind ∼30, ∼26, and ∼28 nt piRNAs, respectively (Girard et al. 2006; Lau et al. 2006; Aravin et al. 2008). Analysis of ping-pong read length combinations shows that the majority of ping-pong pairs combine reads with lengths of ∼26 and ∼30 nt or ∼30 nt both, suggesting that ping-pong occurs primarily between Piwil1 and Piwil2 in a heterotypic manner or homotypically among Piwil1 proteins, but much less between Piwil2 proteins (supplementary fig. S1*D*, Supplementary Material online). Though we note that the sRNAs were not co-immunoprecipitated from PIWI proteins, hence strictly representing piRNA-like RNAs, we will refer to these sequences as piRNAs based on their unambiguous combination of piRNA traits.

### Comparability of Predicted piRNA Clusters Among Individuals and Species

**Fig. 1.**
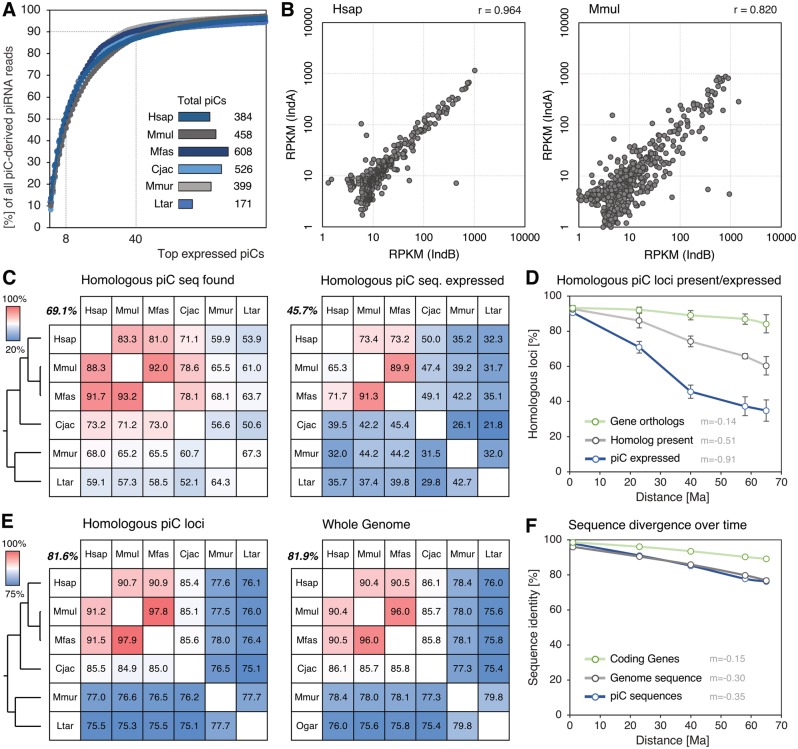
—Comparison of predicted piRNA clusters (piCs) between species and individuals. (*A*) Cumulative distribution of read shares produced by the top 100 expressed piCs and total number of piC loci predicted for each species. (*B*) Correlation of read densities (RPKM) of piCs from two individuals (IndA/B) of the same species (*H. sapiens* and *M. mulatta*). (*C*) Rates of loci for which homologous sequences could be found (left) and rates of homologous loci which are expressed (right). Bold numbers indicate mean percentages; trees show phylogenetic relationships. (*D*) Rates of presence of homologous loci and stably expressed piCs over evolutionary time distances. (*E*) Sequence identity of homologous piC loci (left) and sequence identity of homologous genomic sequence (right). (*F*) Sequence identities over evolutionary time distances.

We identified a varying number of piRNA clusters per species, ranging from 171 to 608 (fig. 1*A*). The majority of reads falls into piRNA clusters except for *L. tardigradus* (supplementary fig. S1*A*, Supplementary Material online), which is likely due to the usage of the *Otolemur garnettii* (northern greater galago) genome for mapping, because a matching reference genome does not exist to this date. Also, in each species a small number of clusters is responsible for the majority of piRNA reads, while the remaining loci account for relatively few reads (fig. 1*A*).

To test whether piRNA cluster predictions are comparable between individuals of the same species and ultimately between different species, we checked the amount of overlap of identified loci based on different sRNA samples. Through our customized approach we could identify nearly all piRNA clusters among individuals of the same species with an overlap of 99.5% of loci in *H. sapiens* and 97.4% in *M. mulatta* (supplementary fig. S2*A*, Supplementary Material online). Furthermore, the expression rate of piRNA clusters, represented by read density, highly correlates between two individuals of the same species, supported by Pearson correlation coefficients of 0.96 for *H. sapiens* and 0.82 for *M. mulatta* (fig. 1*B*, supplementary fig. S2*B*, Supplementary Material online). Together these results show that the expression of piRNA clusters is mostly consistent and comparable between individuals of the same species, making the comparison between different species diagnostically conclusive.

### Presence, Activity and Sequence Evolution of Homologous piRNA Clusters

To determine the proportion of piRNA clusters that are shared among species, we used an approach based on synteny and sequence similarity. Although syntenic regions could be found for nearly all (∼99.4%) piRNA clusters (supplementary fig. S2*C*, Supplementary Material online), the rate of homologous piRNA cluster loci present across species drops substantially the more distantly two species are related, ranging from 93.2% for *M. mulatta* and *M. fascicularis* to 50.6% for *C. jacchus* and *L. tardigradus*/*O. garnettii* (fig. 1*C* and *D*). Furthermore, the proportion of loci that actively produce piRNAs drops even more steeply, as seen when plotted by evolutionary time distance that separates the analyzed species (fig. 1*D*). Nearly all homologous piRNA cluster loci are expressed between *M. mulatta* and *M. fascicularis*, but merely 21.8% of clusters in *C. jacchus* are also active in *L. tardigradus*, which represent only 43% of identified homologous loci. Generally, the rate of loss and gain of piRNA cluster loci is considerably high compared with the amount of orthologous genes that are shared between species (fig. 1*D*).

Overall, for 707 loci homologs were found in every genome, whereas only 156 clusters are consistently expressed across all species. Noteworthily, a previous study described the expression of a core set of 77 piRNA-producing loci that are found throughout eutherians (Chirn et al. 2015). Altogether only 45 of these 77 loci overlap with the 156 consistently expressed piRNA clusters in primates, demonstrating the deactivation of conserved piRNA clusters on specific primate lineages, which tend to produce less piRNAs than the 45 loci that are consistently conserved (supplementary fig. S2*D*, Supplementary Material online).

Our findings suggest that a considerable fraction of primate piRNA clusters is located at genomic regions that are specific to distinct species or lineages, being acquired rather recently on the evolutionary time scale. Indeed, it was previously shown in a study of mouse and rat piRNA clusters that their genomic contexts are very unstable, because many rodent clusters lie within regions that underwent major rearrangements, including insertions, deletions, and inversions (Assis and Kondrashov 2009). The large discrepancy between presence of homologous loci and their actual activity as piRNA clusters indicates that in addition many loci either lost their piRNA-producing activity after their emergence or gained it later after evolutionary partition.

The sequence evolution of homologous piRNA clusters is very similar to the general sequence divergence over time in the whole genome (fig. 1*E*) and is in stark contrast to the relatively slow change of coding-gene sequences (supplementary fig. S2*C*, Supplementary Material online). Comparable with genomic sequence in general, piRNA cluster loci show a near linear decrease in sequence identity over evolutionary times at a roughly doubled rate compared with coding genes (fig. 1*F*), indicating lack of selection pressure on piRNA cluster sequences. This is in line with previous findings which suggested that the small-scale evolution of piRNA clusters proceeds at rates typical for mammalian genomes (Assis and Kondrashov 2009). Lastly, we wondered whether those loci that are consistently expressed in every species might show elevated rates of similarity, however, no consequent substantial shift in any direction could be observed (mean change: −0.33%; standard deviation: 0.3%).

### Adaptation Patterns of Homologous and Nonhomologous piRNA Clusters

**Fig. 2.**
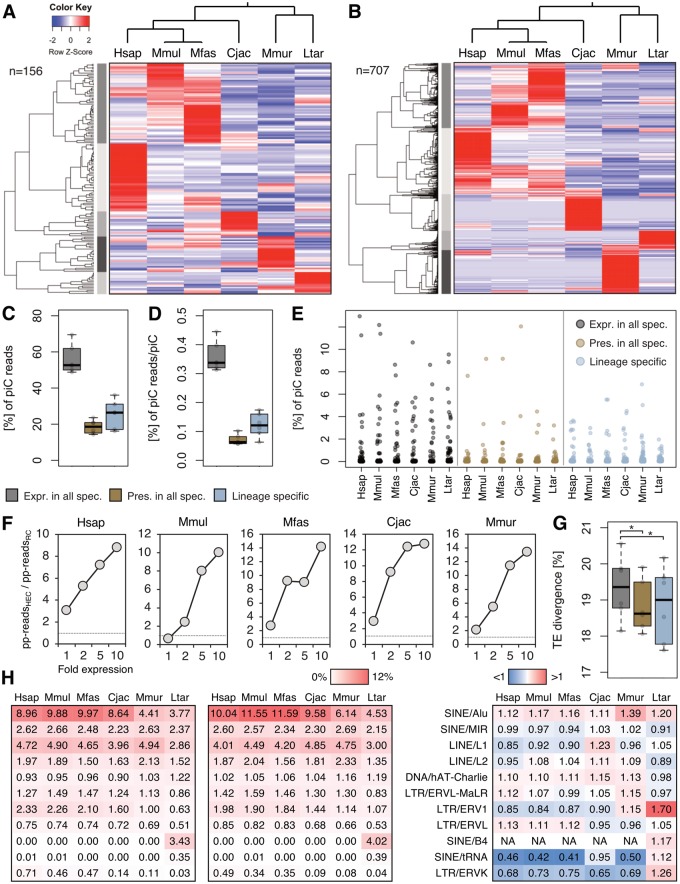
—Adaptations of homologous and nonhomologous piRNA clusters (piCs). (*A*) Differential expression of homologous loci that are consistently expressed in each species. (*B*) Differential expression of homologous loci that are present in each species. Non-expressed loci have an expression value of 0. (*C*) Combined shares of piC-derived piRNA reads per species from piCs that are present and expressed in each species, piCs that have homologs in each genome but not expressed in each species and piCs that do not have homologs in each genome. (*D*) Shares of piC-derived piRNA reads per expressed cluster. (*E*) Shares of reads contributed by each piC to the total pool of piC-derived piRNA reads per species. (*F*) Ratios of shares of reads with ping-pong partner sequences in highly expressed clusters (HEC) with 1-, 2-, 5-, and 10-fold expression compared with all remaining clusters (RC). (*G*) Mean sequence divergences from consensus of transposons in piCs. Same order and key as *C*, *D*. *P <0.05 (paired Wilcoxon–Mann–Whitney test). (*H*) Transposon sequence shares in active piC loci that are ubiquitously expressed (left); transposon sequence shares in active piC loci that are present in every species, but not ubiquitously expressed (mid); ratios of transposon sequence shares between active piC loci that are present in every species, but not ubiquitously expressed and those that are ubiquitously expressed (right).

Next, we analyzed the differential expression of homologous piRNA clusters across species. Loci that are expressed in all species (fig. 2*A*) were examined separately from those that are present in all six genomes, but do not necessarily produce piRNAs (fig. 2*B*). In both cases the expression profiles are very specific for each species, supported by hierarchically clustered dendrograms (fig. 2*A* and *B*; left) which recapitulate the phylogenetic relations of the six primates in a remarkably accurate way (fig. 2*A* and *B*; top).

We then checked the contribution of piRNA clusters with different presence and activity states to the global pool of piRNAs per species. We distinguished clusters that are present and expressed in each species (156/sp., group 1), loci that are found in each genome but are not expressed in every species (∼277/sp., group 2) and those that do not have homologs in each genome (∼222/sp., group 3). Group 1 piRNA clusters contribute the majority of reads across species (50–70%), despite constituting the smallest group. However, although being ubiquitously expressed, their remarkably distinct expression profiles among primates (fig. 2*A*) indicate lineage-specific adaptations of expression rates. The second group provides the smallest amount of reads (14–24%), whereas group 3 clusters contribute slightly larger shares (16–36%) (fig. 2*C*–*E*). Nevertheless, both still produce considerable proportions of piRNA reads, showing that these lineage-specific loci include important contributors to the total piRNA pool.

It is difficult to distinguish pre-pachytene and pachytene piRNA clusters that were predicted from total RNA of adult testis. A previously suggested computational approach defines pre-pachytene and pachytene piRNA clusters depending on whether 26 or 30 nt piRNAs, respectively, are produced in greater amounts at a given locus (Gainetdinov et al. 2017), because these size populations can be attributed to Piwil2 and the pachytene piRNA-specific paralog Piwil1 (Aravin et al. 2008). Using a slightly modified version of this procedure (supplementary fig. S3*A*, Supplementary Material online), we found that across species an average of about 25% of piRNA clusters are 26 nt-leaning, whereas about 75% are 30 nt-leaning, which is in accordance with corresponding shares of total piRNA reads (supplementary fig. S3*B*, Supplementary Material online) and supraprimate adult testis mRNA expression of *Piwil2* and *Piwil1* genes, respectively, though it is slightly shifted toward *Piwil2* (supplementary fig. S3*C*, Supplementary Material online). Similar distributions can be observed for each of the above described differentially conserved cluster groups 1–3 without considerable deviations of the mentioned 25:75 ratio (supplementary fig. S3*D*, Supplementary Material online). Noteworthily, it was proposed that Piwil2 and Piwil1 proteins that both bind pachytene piRNAs compete for the same precursors (Beyret et al. 2012), suggesting that some proportion of 26 nt-leaning loci may nevertheless represent pachytene piRNA clusters.

Because pachytene piRNA cluster expression is initiated by the A-MYB transcription factor (Li et al. 2013) we checked whether there is a difference in presence of A-MYB promoters in homologous piRNA cluster loci that are differentially active between species. Indeed, we found that the A-MYB promoter sequence variant that is most strongly associated with high piRNA read density across species (supplementary fig. S4*A*, Supplementary Material online) is significantly enriched in expressed compared with non-expressed loci, of which homologs are active in other primates (supplementary fig. S4*B*, Supplementary Material online). Furthermore, the highest enrichment is observed in loci with conserved expression across all species. Hence genomic loci presumably gain or lose the ability to produce piRNAs through acquisition or loss of A-MYB promoters.

Furthermore, we looked for differences in piRNA clusters that are highly expressed in a species-specific manner compared with the remaining clusters within the same species to identify adaptations linked to the differential expression of clusters. Although we found no difference regarding the amount of genes, pseudogenes or transposon sequences (measured in % of bp), we noted that the share of reads that possess ping-pong partner sequences consistently grows with increasing species-specific expression (fig. 2*F*). In addition, clusters with at least 10-fold expression in one species compared with the remaining species, produce piRNAs that are enriched for transposon sequences compared with the remaining piRNA transcriptome, which however does not hold true for *C. jacchus* (supplementary fig. S4*C*, Supplementary Material online). Hence, differentially highly expressed piRNA clusters are particularly involved in secondary piRNA biogenesis and thus post-transcriptional regulation, presumably of transposons, as a possible adaptation to species-specific targets.

In support of this notion, we found that the mean transposon divergence from consensus in ubiquitously expressed (group 1) piRNA clusters is significantly higher than in the remaining groups (fig. 2*G*), indicating more recent transposon insertion events in the latter and suggesting that expression of these clusters represents lineage-specific adaptations to newer transposons. We then compared the transposon content of group 1 and group 2 piRNA clusters in more detail (fig. 2*H*). Across all species the primate-specific Alu elements are more abundant in loci with lineage-restricted expression (group 2), whereas other transposon families show differential enrichment. For instance, the transposon types SINE/B4 and SINE/tRNA, which are specifically active in lorisiformes, are enriched in group 2 loci only in piRNA clusters of *L.tardigradus*. These results support our conclusion that loci being expressed in a lineage-specific manner represent an adaptation to recently active transposons, which conforms to the well-established role of piRNA clusters in an anti-transposon arms race (Aravin, Hannon, et al. 2007).

### Characteristics and Evolution of Pseudogenes in piRNA Clusters

**Fig. 3.**
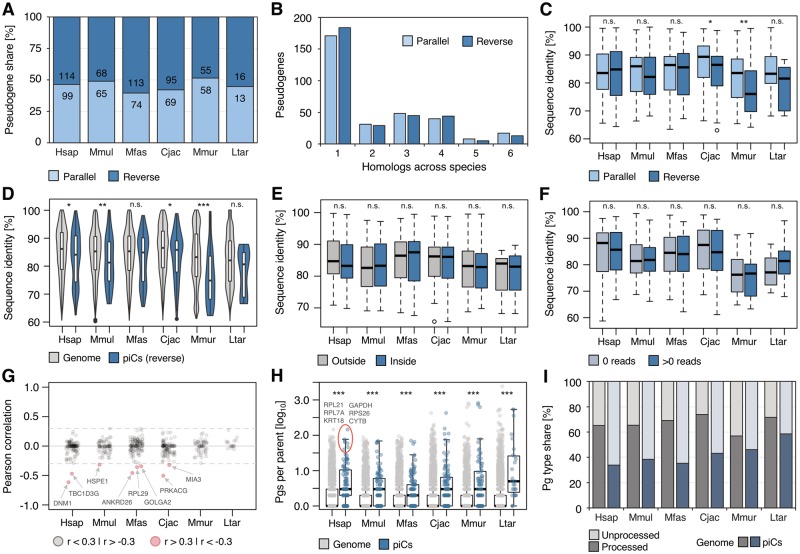
—Characterization of pseudogenes in piRNA clusters (piCs). (*A*) Number of pseudogenes sorted by parallel or reverse orientation relative to piC directionality. (*B*) Number of pseudogene homologs in homologous piC loci in parallel and reverse orientation shared among species. (*C*) Sequence identities to parent genes of piC pseudogenes in parallel compared with reverse orientation. (*D*) Sequence identities to parent genes of reverse pseudogene sequences in piCs compared with pseudogenes of whole genomes. (*E*) Sequence identities to parent genes of parts of reverse pseudogenes in piCs compared with corresponding parts outside of piCs. (*F*) Sequence identities to parent genes of 32 nt windows of reverse pseudogene sequences in piCs that produce antisense piRNAs compared with 32 nt windows of the same pseudogenes that show no piRNA production. (*G*) Pearson correlations of sequence identity to parent gene and produced antisense piRNA read density for each reverse pseudogene in piCs. Correlations between −0.3 and 0.3 (broken lines) are considered negligible. (*H*) Total (genome-wide) numbers (log10) of pseudogenes (Pgs) per parent gene of pseudogenes in piCs and whole genomes. (*I*) Shares of processed and unprocessed pseudogenes in whole genomes and all piCs. *P < 0.05; **P < 0.01; ***P < 0.001; n.s.: *P* > 0.05 (Wilcoxon–Mann–Whitney test: *C*, *D*, *H*; paired Wilcoxon–Mann–Whitney test: *E*, *F*).

To gain a deeper understanding of possibly shared attributes of pseudogenes that lie in piRNA clusters, we set out to determine their basic properties in each species. First, to discern if a specific piRNA cluster type is enriched for pseudogenes, we checked whether the ratio of 26 nt- to 30 nt-leaning clusters containing pseudogenes compared with loci without pseudogene sequences differs from the observed 25:75 for all clusters, however no significant difference could be detected (supplementary fig. S3*E*, Supplementary Material online). Because reverse orientation of pseudogene sequences relative to piRNA cluster directionality is a prerequisite for the generation of gene-targeting antisense piRNAs, we next assessed the shares for each condition. We noticed a slight bias for reverse orientation of pseudogenes, which however is not consistent across all species (fig. 3*A*) and overall not statistically significant (paired Welch *t*-test, *P* = 0.09). To test the possibility that the insertion of pseudogenes might not occur equally frequent in both directions, for example that the probability of parallel insertion might be underestimated, we examined the genome-wide presence of pseudogene copies in introns. This analysis showed that in introns of both, coding-genes and long noncoding RNAs (lncRNAs), pseudogenes show a tendency toward reverse orientation relative to gene- or lncRNA-transcription direction with respectively 59% and 57% reverse copies in human and an average of 58% for gene introns across primates (standard deviation: 3%), supporting the former result. Another prediction, based on the assumption that pseudogene-derived gene targeting by piRNAs provides an evolutionary benefit, is a higher retention rate of pseudogenes in piRNA clusters in reverse orientation than in parallel. However, the observed amounts of homologous pseudogenes of both orientations are very similar, regardless of how many species share the corresponding copies in homologous piRNA clusters (fig. 3*B*).

We then asked whether reverse pseudogenes are more similar to their parent genes, because a high degree of sequence similarity is required for piRNA target recognition (Reuter et al. 2011; Huang et al. 2013), which would be expected if pseudogene-derived gene targeting by piRNAs is conserved. However, no elevated sequence similarity of reverse pseudogenes compared with those in parallel orientation could be observed, but instead the opposite is the case in two species (fig. 3*C*). The same analysis, comparing reverse pseudogenes in piRNA clusters and all pseudogenes in the whole genome, shows no completely consistent pattern, though in four species a statistically relevant bias toward even lower sequence similarity of reverse pseudogenes in clusters to their parent genes can be observed and the tendency is similar for the two remaining species (fig. 3*D*). Higher sequence divergence can indicate older age of these pseudogenes, but it is also conceivable that an unwanted interference with normal gene expression by pseudogenic piRNAs might result in increased sequence evolution of the corresponding pseudogenes as a means to escape piRNA targeting, as found for miRNAs (Farh et al. 2005). To test this hypothesis, we compared the sequence divergence of the parts of reverse pseudogenes that overlap with piRNA clusters to any parts of the same copies that lie outside of these clusters, however no difference could be detected (fig. 3*E*). Because not every possible piRNA-sized region of reverse pseudogenes actually produces piRNAs, we scanned these sequences with a 32 nt sliding window and compared the divergence of sites producing piRNA reads to those without reads, yielding similar results (fig. 3*F*). Using the same approach, we then determined the correlation of sequence similarity to parent gene with read density for each reverse pseudogene and found a negative correlation (*r* < −0.3) for some but no positive correlation (*r* > 0.3) in any case (fig. 3*G*). This indicates a weak influence of piRNA production on sequence evolution for most pseudogenes but a possible accelerated evolution for a few sequences. The parents of the latter tend to be involved in basic cellular processes such as vesicle formation (DNM1, GOLGA2, MIA3), and represent widely used types like heat-shock (HSPE1) or ribosomal (RPL29) proteins.

Looking closer at parental genes in general, we noticed a tendency toward an increased number of pseudogene offspring compared with genome-wide parental genes (fig. 3*H*), whereas there is no difference between parents of parallel and reverse copies (supplementary fig. S5*A*, Supplementary Material online). Because housekeeping genes are known to have numerous pseudogenes (Kalyana-Sundaram et al. 2012), we examined the shares of human housekeeping genes using a previously described method based on expression consistency across tissues (Eisenberg and Levanon 2013) and found that 28% of reverse pseudogene parents (29% overall) fall in this category, which applies to only 20% of all genes. Together, these results demonstrate that in general reverse pseudogenes in piRNA clusters show no signs of selection pressure and do not exhibit the traits that would be expected if pseudogene-derived piRNAs were widely used for regulation of coding genes.

Previously, it was suggested that piRNA clusters may gain the ability to target coding genes through the integration of gene transcripts by retrotransposition, resulting in the formation of processed pseudogenes (Hirano et al. 2014; Gebert et al. 2015). However, our analysis of pseudogene types shows that whereas processed pseudogenes vastly outnumber unprocessed copies in primate genomes, which is in line with previous studies (Sisu et al. 2014), this relation consistently shifts toward unprocessed pseudogenes in piRNA clusters (paired Welch *t*-test, *P* = 0.0019) (fig. 3*I*). This indicates that retrotransposition is likely not a main contributor for the incorporation of pseudogenes into piRNA clusters. Because it has been shown in rodents that many piRNA clusters originate through duplication by ectopic recombination (Assis and Kondrashov 2009), it could be speculated that genes which accidentally overlap with clusters might get duplicated together with the piRNA-producing locus and then undergo pseudogenization, thus being a byproduct of piRNA cluster emergence.

### Gene Targeting of Pseudogene-Derived piRNAs within and Across Species

**Fig. 4.**
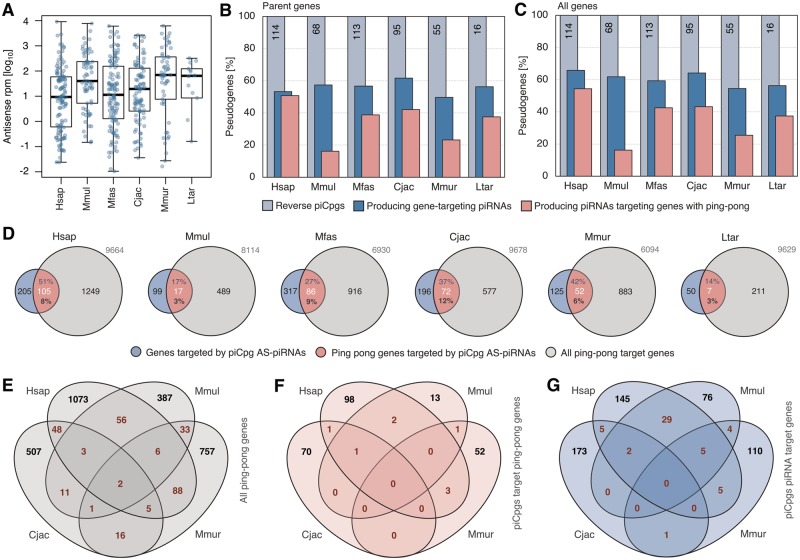
—Targeting of protein-coding genes by pseudogene-derived piRNAs. (*A*) piRNA reads (in log10 rpm) derived from reverse pseudogenes in piRNA clusters (piCs). (*B*) Amounts of reversed pseudogenes in piCs (piCpgs) that produce antisense piRNAs against parent genes and those producing antisense piRNAs targeting parent genes with ping-pong signature. (*C*) Amounts of reversed pseudogenes in piRNA clusters that produce antisense piRNAs against any genes and those producing antisense piRNAs targeting any genes with ping-pong signature. (*D*) Shares of genes targeted by pseudogene-derived antisense (AS) piRNAs that exhibit ping-pong signatures compared with all ping-pong genes. Total number of all genes with piRNA targeting shown in gray. (*E*) Homology of ping-pong target genes among human, macaque, marmoset, and mouse lemur. (*F*) Homology of ping-pong target genes targeted by pseudogene-derived antisense piRNAs among human, macaque, marmoset, and mouse lemur. (*G*) Homologous genes with piRNA coverage, targeted by pseudogene-derived antisense piRNAs, among human, macaque, marmoset, and mouse lemur.

In the next step we examined the gene-targeting capacities of piRNA cluster-overlapping pseudogenes. In each species reverse pseudogenes produce a highly variable quantity of piRNA reads with median values of about 9–70 reads per million (rpm) (fig. 4*A*). Furthermore, we found that on average 56% of reverse pseudogenes located in clusters give rise to piRNAs that potentially target their parent genes, however only a mean of 36% of reverse pseudogenes produce piRNAs that aim at parent genes with ping-pong signatures (fig. 4*B*). Considering the targeting of coding genes in general, these shares increase only slightly to 60% and 38%, respectively (fig. 4*C*). This indicates that a large part of pseudogenes in piRNA clusters is ineffective in triggering piRNA-dependent processing of putative target genes.

Next, we set the portion of protein-coding genes that are potentially targeted by pseudogene-derived piRNAs in relation to all target genes that show a significant ping-pong signature (referred to as ping-pong genes in the following) (fig. 4*D*). Overall, on average 31% of genes targeted by pseudogene-derived antisense piRNAs also showed a ping-pong signature. However, only small fractions of genes with ping-pong signatures were targeted by pseudogene-derived antisense piRNAs. We note that confining this analysis to testis-expressed genes data, which however is not available for all species, reduces the overall number of target genes by about 10%. Because the targeting of coding genes by piRNAs derived from pseudogenes lying in piRNA clusters cannot explain the vast majority of cases of ping-pong coverage on gene transcripts, other mechanisms that initiate processing by the secondary piRNA pathway on protein-coding genes probably play a far greater role. Nevertheless, the fact that still a part of the genes that are targeted by pseudogene-derived piRNAs indeed display a ping-pong signature, shows that some of these piRNAs likely have the expected capability to lead gene transcripts into the ping-pong cycle.

Examining the evolutionary relationships of ping-pong genes in general among primates, we found that for the vast majority of genes ping-pong processing is lineage specific, whereas only a limited number of homologs is targeted in multiple species (fig. 4*E*). Restricting this analysis to ping-pong genes that are targeted by pseudogene-derived antisense piRNAs yields markedly less overlap between target gene homologs. Not a single homologous target is shared among four species and only one gene shows a ping-pong signature in three species, namely human, macaque, and marmoset (fig. 4*F*). Even when expanding the circle of potential homologous targets to genes showing general piRNA coverage above 5 reads per kilo base per million (RPKM), regardless of a presence of ping-pong signatures, the amount of orthologous targets remains very limited (fig. 4*G*).

Together these results suggest that the PIWI/piRNA pathway triggered by pseudogene-derived antisense piRNAs, is either evolutionary highly variable or alternatively of lesser relevance for the regulation of genes in most cases. Importantly, it was shown in mice that the knockdown of a piRNA cluster containing a pseudogene did not lead to any phenotypic effect such as a deficiency or impairment, although the expression level of the corresponding parent gene did in fact change (Watanabe et al. 2015). Thus it appears likely that, whereas the presence of pseudogenes in piRNA clusters in reverse orientation has the potential to affect gene targeting, the consequences on the regulation of these genes is usually not as pronounced as to have such a strong physiological relevance that it would be maintained over evolutionary times. However, we cannot rule out that in some cases such a system might indeed have a crucial function.

Considering gene-targeting by piRNAs on a global scale, we found that whereas the total amount of homologous ping-pong genes is rather low (fig. 4*E*), the number of gene homologs with piRNA coverage above 5 RPKM throughout species in general, with overall 1,428, is considerably higher (supplementary fig. S5*B*, Supplementary Material online). Gene ontology analysis with this gene set indicates enrichment in a variety of functions, localizations and processes, including spermatogenesis, translation regulation, mRNA processing, and oxidative phosphorylation (supplementary tables S1–S3, Supplementary Material online). Furthermore, we checked whether there is a bias toward testis-enriched genes, however the corresponding share in this gene set (4.81%) is not significantly different from that of all testis-expressed genes (5.16%) in human (*P* = 0.617, Fisher exact test). Generally, on average a majority of 75.8% of genic reads derives from sense strands in each species. Because longer 3′-UTRs can harbor more transposon sequences as potential piRNA target sites, we tested whether there is a relationship between piRNA read coverage and 3′-UTR length in human. Although we found no correlation within target genes regarding RPKM (Pearson’s *r* = 0.0005), target genes in general have on average longer 3′-UTRs than non-targets (means: 2,027 and 1,478 bp; medians: 1,346 and 858 bp). Wondering what differentiates ping-pong genes from other genes with piRNA coverage, we checked whether there is a relationship between read coverage and the probability for showing a ping-pong signature. Indeed, we found a strong correlation for increased shares of ping-pong targeting among genes within greater RPKM ranges in each species (Pearson’s *r* = 0.86–0.98) (supplementary fig. S5*C*, Supplementary Material online). This suggests that the ping-pong cycle might play a role in regulating some genes that are consequently more heavily processed.

The considerably large set of genes that exhibits piRNA coverage in all analyzed species indicates a conserved mechanism for PIWI-mediated gene regulation which however is independent of pseudogene-derived piRNAs. Noteworthily, the targeting of protein-coding genes by piRNAs seems to be widespread in animals and evolutionary ancient because it is found not only in mammals but also in many protostomians (Jehn et al. 2018; Lewis et al. 2018) and cnidarians (Juliano et al. 2014; Modepalli et al. 2018) and in both taxa even in somatic tissues. It was shown that 3′-UTRs exhibit the greatest sense piRNA read density on coding genes in diverse metazoan lineages (Robine et al. 2009; Ha et al. 2014) and later it was demonstrated that transposon sequences that reside in 3′-UTRs can be targeted by piRNAs, which presumably leads to mRNA decay (Watanabe et al. 2015). Another study showed that the piRNA production from some genes, partly overlapping with our set of homologous genes (29 out of 57 genes), is conserved in eutherians (Chirn et al. 2015). One of these genes, namely CBL, was recently demonstrated, among others, to be repressed by Aub-bound piRNAs in the germline of *Drosophila* through translational repression by binding at 5′- and 3′-UTRs, particularly at transposon insertion sites (Barckmann et al. 2015; Rojas-Ríos et al. 2017). Thus, some genes are apparently targeted in a highly conserved manner. Moreover, it was shown in mice that pachytene piRNAs induce broad mRNA elimination in mouse elongating spermatids by recruiting the deadenylase CAF1 upon recognition of target sites, which are mainly located in 3′-UTRs (Gou et al. 2014). Hence, more than pseudogene-derived sequences, transposon-associated piRNAs are presumably the major regulators for PIWI-dependent processing of protein-coding genes. This, however, raises the question of why mammalian piRNA clusters are enriched for pseudogene sequences anyway.

### Genomic Environments of piRNA Clusters

**Fig. 5.**
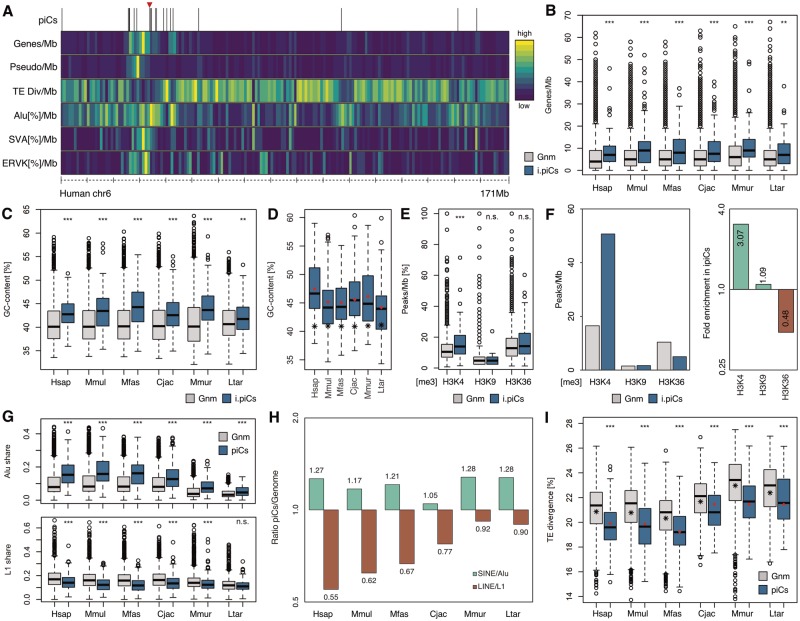
—Genomic environments of piRNA clusters (piCs) in primates. (*A*) Heatmap showing human chromosome 6 in 1 Mb slices. piCs: piC locations; Genes/Mb: Gene density; Pseudo/Mb: Pseudogene density; transposon div/Mb: Mean transposon divergence per Mb; transposon[%]/Mb: Total shares of Alu, SVA, and ERVK elements per Mb. The large, highly expressed and conserved piC at chr6:33,863,000-33,927,000 is marked by an arrow. Genomic environments of piCs in primates. (*B*) Gene densities of 1 Mb genomic slices that contain intergenic piCs compared with the whole genome. i.piC: intergenic piCs; Gnm: whole genome. (*C*) GC contents of 1 Mb genomic slices that contain intergenic piCs compared with the whole genome. (*D*) GC contents of intergenic piCs. Total means of all intergenic piC sequences per species are indicated by red diamond shaped points. Means of whole genomes are shown by star shaped points. (*E*) Peak densities for histone modifications H3K4me3, H3K9me3, H3K36me3 of 1 Mb genomic slices that contain intergenic piCs compared with the whole genome in human. (*F*) Mean peak densities for histone modifications H3K4me3, H3K9me3, H3K36me3 in genome and in intergenic piCs (left) and ratios (right). (*G*) Total Alu/L1 shares of 1 Mb genomic slices that contain piCs compared with the whole genome. (*H*) Ratios of Alu/L1 sequence shares between piCs and genomic sequence. (*I*) Mean transposon divergences of 1 Mb genomic slices that contain piCs compared with the whole genome. Total means of all piC sequences per species are indicated by red diamond shaped points. Means of whole genomes are shown by star shaped points. **P < 0.01; ***P < 0.001; n.s.: *P* > 0.05 (Wilcoxon–Mann–Whitney test).

As most pseudogenes in piRNA-producing loci do not to play a role in gene regulation, we looked for potential alternative explanations for the enrichment of pseudogenes in piRNA clusters and turned our attention to their genomic environments. We scanned the primate genomes with a resolution of 1 million base pairs (Mb) to obtain information on gene and pseudogene density, shares of different transposon families and total sequence divergence of transposons. First, we noticed that piRNA clusters tend to be located in gene rich regions, as seen for instance on human chromosome 6 (fig. 5*A*), which in a particular gene-dense region carries one of the largest and most strongly expressed piRNA clusters that is active across all six analyzed primates and additionally in tree shrew and mouse (Goh et al. 2015; Rosenkranz, Rudloff, et al. 2015). Analyzing the complete human genome, ignoring centromeric regions, we found that piRNA clusters indeed show a significant tendency to be located in genomic regions with elevated gene density, compared with the whole genome (supplementary fig. S6*A*, Supplementary Material online). This holds also true if solely loci containing neither genes nor pseudogenes, hence being completely intergenic, are considered, which is the case for all six primate species (fig. 5*B*). Besides, 26 nt- and 30 nt-leaning clusters show no statistically significant difference in this regard (supplementary fig. S3*F*, Supplementary Material online).

Furthermore, we noticed that regions in which intergenic piRNA clusters are located show elevated percentages of guanine and cytosine (GC) bases (fig. 5*C*). Also, the GC content of intergenic piRNA clusters themselves is on average higher than the genome-wide rate across species (fig. 5*D*). Gene density is known to be correlated with open chromatin structure (Gilbert et al. 2004) and GC rich regions tend to indicate a more active chromatin conformation (Dekker 2007). In support, using ChIP-seq data from human testis we found that the genomic context of intergenic piRNA clusters is significantly biased toward the activating histone modification H3K4me3 (Lawrence et al. 2016), in contrast to the repressive H3K9me3 and ambiguous H3K36me3 (Chantalat et al. 2011) modifications (fig. 5*E*). Furthermore, intergenic piRNA clusters themselves are highly enriched for H3K4me3 (3.07-fold), while being depleted of H3K36me3 (0.48-fold) and neither considerably enriched for nor depleted of H3K9me3 marks (1.09-fold) compared with the whole genome (fig. 5*F*). These results indicate that primate piRNA clusters represent euchromatic regions within largely euchromatic genomic contexts.

Several other factors correlate with gene abundance. First of all, unsurprisingly, gene density is also correlated with pseudogene abundance per Mb (supplementary fig. S6*B*, Supplementary Material online), which is similar in all analyzed species (Pearson’s *r* = 0.314–0.369, *P* < 0.001). Furthermore, there is a significant negative correlation with transposon divergence, suggesting that younger transposons are enriched in gene-rich regions (supplementary fig. S6*B*, Supplementary Material online). Correspondingly, both, the primate-specific Alu elements (Kriegs et al. 2007), as well as the hominid-specific SVA family elements (Wang et al. 2005) tend to be more abundant in gene-rich regions of the human genome. However, the share of L1 elements tends to be increased in gene-poorer segments. This pattern, particularly of Alu and L1 transposons with respect to gene-density was already noticed in the first analysis of the human genome sequence (Lander et al. 2001).

We analyzed whether the respective positive and negative correlations of Alu and L1 element abundance with gene density leads to a bias of cluster localization with regards to shares of Alu and L1 transposons. Indeed, we found that piRNA clusters show a significant tendency for regions with higher share of Alu elements, relative to the whole genome, whereas the opposite is true for L1 transposons, though less distinctly (fig. 5*G*). Correspondingly, piRNA clusters are depleted of L1 and enriched for Alu elements across primate species (fig. 5*H*). Furthermore, piRNA clusters show a significant bias for regions with lower average transposon divergence, relative to the whole genome (fig. 5*I*), which is an indication of younger transposon age and hence more recent transposition. The decreased amount of L1 sequences in piRNA clusters might be explained by the fact that these transposons have emerged much earlier than Alus and that correspondingly only a minority of copies is still active in the genome (Beck et al. 2010). Despite a reduced share of L1 elements in piRNA clusters, however, corresponding piRNAs are abundantly present in each species (supplementary fig. S7, Supplementary Material online), which is in agreement with findings in mouse, showing that L1 transposons are major targets of pachytene piRNAs (Reuter et al. 2011).

Finally, we note that the above described findings are not primate-specific because analysis of adult testis-expressed mouse piRNAs yields similar results regarding the genomic contexts of piRNA clusters (supplementary fig. S6*C*, Supplementary Material online) with respect to gene density, GC content and shares of L1 transposons and rodent B1 elements, which share a common ancestor with primate Alu elements (Kriegs et al. 2007).

Together, these results suggest that mammalian piRNA clusters are more likely to inhabit more active regions of the genome with a more open chromatin structure. While *Drosophila* piRNA clusters exhibit heterochromatic features (Brennecke et al. 2007), it has been demonstrated in BmN4 cells that piRNA clusters of the silkworm are enriched with euchromatic epigenetic marks, foremost H3K4me3 and H3K4me2 (Kawaoka et al. 2013). Also, mouse pachytene piRNA clusters were shown to be transcribed from H3K4me3-enriched promoters (Li et al. 2013). Our results indicate that, rather than representing islands of open chromatin within heterochromatic regions, mammalian piRNA clusters are embedded in active regions of the genome that are more likely to contain newer transposon copies as well as pseudogene sequences in higher abundance. This might at least in part explain the frequent presence of pseudogenes in mammalian piRNA clusters.

## Conclusion

Pseudogenes that are located in piRNA clusters in reverse orientation have been suggested to be an important source of pachytene antisense piRNAs that direct regulation of parent genes (Hirano et al. 2014; Gebert et al. 2015). However, due to a lack of evidence for selection and very weak conservation of targeting of homologous genes, our study indicates that the presence of pseudogenes in piRNA-producing loci might largely represent a byproduct of piRNA cluster emergence in active genomic regions and in most cases does not have a significant impact on gene regulation by pachytene piRNAs, which nevertheless must not be true for all such instances. Instead, piRNA targeting of transposons in 3′-UTRs (Watanabe et al. 2015; Zhang et al. 2015) is more likely to represent the main mode of gene regulation in late mammalian spermatogenesis. However, further research is needed to validate this claim. For instance, one would expect that certain transposon sequences in the 3′-UTRs of some genes become evolutionary fixed and conserved to ensure faithful regulation by piRNAs, whereas other transposon insertions would presumably have a negative effect, due to unwanted interference with normal gene expression. The examination of such signs of selection and conservation of transposons in 3′-UTRs would help to understand the mechanisms and the evolution of piRNA-mediated gene regulation. Finally, although this study provides insights into the evolution of mammalian pachytene piRNA clusters, it is still difficult to state what the exact forces are that drive piRNA cluster evolution and ultimately what circumstances transform a silent genomic locus into a new, active piRNA cluster.

## Materials and Methods

### Small RNA Library Preparation and Sequencing

Total RNA was isolated from adult testis tissue of *M. murinus* and *L. tardigradus* with TRI Reagent (Sigma-Aldrich). Isolated RNA was applied to a 12% urea-based denaturing acrylamide gel and run for 20 min at 1,200 V and 50 mA (60W) together with a 31-mer internal RNA marker piSPIKETM (IDT) and GeneRuler Ultra Low Range DNA ladder (ThermoScientific). The small RNA fraction ranging from 20 to 35 nt was excised from the gel and dissolved in H_2_O with the Ultrafree-MC system (Millipore). The RNA eluate was desalted using the Amicon Ultra 3 K system (Millipore). A 5′-activated and 3′-blocked RNA adapter (5′-AppCUGUAGGCACCAUCAAUddC-3′) was ligated to the 3′ end of the isolated RNA in absence of ATP to avoid sRNA multimerization. The ligation product was purified via ethanol precipitation and separated from un-ligated adapter and sRNA molecules by PAGE applying a 48-mer RNA size marker. The ligation product was excised from the gel, dissolved, concentrated, and desalted as described above but using an Amicon Ultra 10 K instead of an Amicon Ultra 3 K filter device. Following the ligation of a second RNA adapter to the 5′ end of the RNA (5′-GACUGGAGCACGAGGACACUGACAUGGACUGAAGGAGUAGAAA-3′), RNA was purified via ethanol precipitation, reverse-transcribed with Invitrogen’s Superscript III RT system (reverse primer: 5′-CTGTAGGCACCATCAAT-3′) and PCR amplified (forward primer: 5′-ACATGGACTGAAGGAGTAGA-3′, reverse primer: 5′-CTGTAGGCACCATCAAT-3′) according to the following thermal cycling profile: 5′ at 95 °C, [30″ at 95 °C, 30″ at 51 °C, 20″ at 74 °C] × 11, 5′ at 74 °C. All steps were conducted according to the corresponding manufacturer’s instructions. Sequencing was performed on an Illumina HiSeq 2000 machine.

### Small RNA Data Sets and Basic Analysis

Testis-expressed small RNA transcriptome data sets from adult haplorrhine primates were obtained from NCBI’s sequence read archive (SRA) (ncbi.nlm.nih.gov/sra), including samples from *H. sapiens* (SRR835325), *M. mulatta* (SRR116839), *M. fascicularis* (SRR1755243), and *C. jacchus* (SRR1041905), and for comparisons within species, additional data sets for *H. sapiens* (SRR835324) and *M. mulatta* (SRR553581) were used (supplementary table S4, Supplementary Material online).

Adapter clipping, filtering of low complexity reads and removal of annotated ncRNAs was achieved with unitas (v1.4.6) (Gebert et al. 2017), using default settings. Subsequently, the cleaned sRNA reads were mapped to the corresponding genomic sequences (GRChg38, rheMac8, macFas5, calJac5, micMur3) with the tool sRNAmapper (v1.0) (Roovers et al. 2015; Rosenkranz, Han, et al. 2015), retaining only the best matches (option “-a best”). Because there is no sequenced genome available for *L. tardigradus*, the genome of the closest relative at hand, *O. garnettii* (otoGar3), was used instead. Genome sequences were obtained from the UCSC genome server (hgdownload.cse.ucsc.edu/downloads.html). Basic analyses of sRNA data sets, aimed at the inspection of piRNA characteristics, such as read length distribution, positional nucleotide composition and rates of 5′ overlap lengths, were performed using ngs toolbox (Roovers et al. 2015; Rosenkranz, Han, et al. 2015). Generally, the quantity of reads mapping to multiple sites was fractionated for each sequence by the total number of hits it produced (reads/hits). Furthermore, the analysis of ping-pong partners was carried out, as the majority of the following analyses, using in-house perl scripts (supplementary table S5, Supplementary Material online).

### Prediction of piRNA Clusters

For in-silico prediction of piRNA clusters, we used proTRAC (v2.4.0) (Rosenkranz and Zischler 2012), where two different approaches were used for each species, using a strict and less strict set of options. First, piRNA clusters were predicted with a minimum cluster size of 5 kb (option “-clsize 5000”), a *P* value for minimum read density of 0.01 (option “-pdens 0.01”), a minimum fraction of normalized reads that have 1T (1U) or 10A of 0.75 (option “-1Tor10A 0.75”) and rejecting loci if the top 1% of reads account for more than 90% of the normalized piRNA cluster read counts (option “-distr 1-90”). In a less stringent procedure, we changed the options to a minimum cluster size of 2.5 kb, a *P* value for minimum read density of 0.05 and a minimum fraction of normalized reads that have 1T (1U) or 10A of 0.5. Further setting that depart from the default include a minimal fraction of hits with 1T (U) and 10A of 0.33 (option “-1Tand10A 0.33”), minimal fraction of hits on the main strand of 0.5 (option “-clstrand 0.5”). Generally, proTRAC input included a file containing mapped reads, the corresponding genome sequence file, a repeatmasker annotation file, obtained from the UCSC genome server and a GTF gene annotation file taken from Ensembl (ensembl.org/info/data/ftp/index.html). Finally, neighboring clusters with a distance less than 10 kb were merged. For comparison of piRNA clusters between individuals of the same species, genomic locations and read densities (reads/kb) were extracted from proTRAC output generated with strict options and with less stringent options (supplementary methods, Supplementary Material online). Pachytene (30 nt-leaning) and pre-pachytene (26 nt-leaning) piRNA clusters were distinguished depending on whether the fractions of 25–27 or 29–31 nt piRNAs are greater at a given locus, similarly to the approach described by Gainetdinov et al. (2017).

### Identification of Homologous piRNA Clusters

The bioinformatic procedure for the identification of homologous piRNA clusters between primate species was divided into three main subsequent steps, based on loci predicted with strict proTRAC options. First, exons of the ten flanking protein-coding genes up- and downstream of piRNA clusters were localized in the query genome using GFF gene annotation data and extracted from the genomic sequence.

Next, to find the corresponding syntenic regions, we scanned the repeatmasked subject species genomes for sequences homologous to the flanking gene exons of the respective query species, using the blastn command line tool from the NCBI BLAST+ suite (v2.7.1+) (Camacho et al. 2009). Neighboring BLAST hits were grouped to contiguous gene loci, which in turn were divided into putative syntenic flanks. The most probable syntenic regions were selected according to the number of homologous genes and their sequence similarity to the query genes. Regions with less than four homologous genes were rejected.

If a syntenic region was found, we screened it for sequence homology to the respective query species piRNA cluster, using the discontiguous-megablast algorithm (blastn run with option “-task dc-megablast”), because the sequence conservation of piRNA clusters is expected to be lower compared with protein-coding genes (Assis and Kondrashov 2009). The resulting BLAST hits were then sorted, grouped and ranked according to alignment length, genomic region size, and query coverage. Hit groups falling below thresholds for query coverage (5%), alignment length (1.5 kb), or relative size (15%) were discarded. Finally, to reconstruct the evolutionary relationships of homologous piRNA clusters among six primate species examined in this study, we combined pairs of homologous loci to chains between species.

### Analysis of Homologous piRNA Clusters

For the analysis of sequence conservation and presence/absence status of piRNA cluster loci between species, we extracted the relevant information from BLAST alignment data and compared mean identities and total shares of loci for which a homologous sequence was found for each combination of species. In addition, mean sequence similarities of exonic sequences between species were obtained using discontiguous-megablast on CDS files from NCBI’s genome resource, extracting identities from alignments of gene homologs. The same approach was used to get sequence similarities of genomic sequence, based on comparison between masked chromosomes or contigs homologous to human chromosome 1. To inspect which homologous piRNA cluster loci were actually expressed, we checked if an identified homologous locus was predicted as a piRNA cluster by proTRAC in a less strict mode.

Subsequently, the corresponding data were sorted by the time that had passed since the split of the respective species. The time distance between the two Macaque species *M. mulatta* and *M. fascicularis* was set to about 1 million years (Li et al. 2009). Furthermore, the Split of hominoidea and cercopithecoidea is estimated at 25 million years ago (Ma) (Stevens et al. 2013), whereas catarrhine and platyrrhine primates are thought to have split 40 Ma (Shumaker and Beck 2003). Finally, haplorrhines and strepsirrhines diverged about 65 Ma (Birx 2006), whereas within the strepsirrhines, lemuriformes, and lorisiformes split about 58 Ma (Masters et al. 2012).

Differential expression analyses of homologous piRNA clusters between different species were performed using hierarchical clustering, average linkage, and Pearson distance. Read counts (reads per million, rpm) were extracted from proTRAC output and plotted as contributions to the pool of cluster-derived reads. Transposon divergence rates for each group were extracted from Repeatmasker output and plotted as mean transposon divergence for each species. All statistical testing was performed using R (v3.4.3) and Rstudio (1.1.414) packages.

### Prediction of Pseudogenes

Because the quality of available pseudogene annotations varies substantially among species, for example for GFF data from NCBI (supplementary fig. S8, Supplementary Material online), a custom pseudogene prediction routine was applied, based on the method used by Gerstein and colleagues (Zhang et al. 2006; Sisu et al. 2014). The procedure begins with the search for sequences with similarity to known protein-coding genes in the corresponding repeatmasked genome, using discontiguous-megablast (Camacho et al. 2009) with CDS data, obtained from NCBI’s genome resource, as query sequences. BLAST hits that overlap with gene exons in GFF gene annotation were discarded.

Next, overlapping hits were merged to form larger structures, which in turn were combined with adjacent hits to assemble pseudogene units if the genomic distance did not exceed a threshold that was calculated for each putative pseudogene/parent combination as the 1.5-fold of the largest parent gene intron size, but it was not allowed to fall below 30 kb.

In the third step, the most probable parent genes for the presumed pseudogene loci were selected, based on sequence identity, the best *e*-value of the original BLAST hits and the overall query coverage. In addition, short isolated fragments (<300 bp length or <10% query coverage) were discarded. Lastly, the predicted pseudogene units were classified as processed or unprocessed pseudogenes, depending on their number of pseudo-exons compared with the number of exons of their parent genes and the overall query coverage. Specifically, if the number of predicted pseudo-exons was half the number of expected pseudo-exons (coverage fraction times number of parent exons) or less, it was categorized as a processed pseudogene.

### Analysis of piRNA Cluster Pseudogenes and Identification of Homologs

Sequence identities of pseudogenes to parent genes, information on orientation with respect to directions of piRNA cluster transcription, as well as shares of processed and unprocessed pseudogenes were extracted from BLAST alignment output and our custom pseudogene annotation. Insertion rates of pseudogenes in gene introns were determined using GFF gene annotation data. To determine which pseudogenes are present throughout homologous piRNA clusters across species, for each pseudogene sequence that is located in a cluster locus a similar sequence was searched for in any homologous locus that was previously identified, using discontiguous-megablast, while filtering out short total alignments (<150 bp) and hits with coverage below 30%. Analysis of human housekeeping genes was performed using expression data accessed from the EMBL-EBI Expression Atlas database (ebi.ac.uk/gxa, Petryszak et al. 2016) with the methodology described in Eisenberg and Levanon (2013).

### Prediction of piRNA Target Genes

To identify piRNA targets among protein-coding genes, clean reads with a length between 24 and 32 nt were mapped to known coding cDNA sequences, obtained from Ensembl, in each species, using seqmap (Jiang and Wong 2008). Two mismatches were allowed during mapping and the output was subsequently filtered to permit two mismatches in antisense but none in sense orientation. For the read coverage, a threshold of 5 RPKM per gene was applied. A significant ping-pong signature was declared being present if the largest number of overlaps was unambiguously 10 nt long and in addition if the *z*-score for 10 nt overlaps compared with the background (1–9 and 11–20 nt overlaps) was greater than *z* = 2.3264, corresponding to a *P* value of less than *P* = 0.01 (Zhang et al. 2011). For limiting these analyses to testis-expressed genes we used expression data from the EMBL-EBI Expression Atlas database (Petryszak et al. 2016) and applied a threshold of 0.5 transcripts per million, used by the database.

To find potential gene targets of antisense piRNAs derived from pseudogenes, reads that match the opposite strands of reversed pseudogenic regions in piRNA clusters were mapped to the coding subset of known cDNA sequences with seqmap (Jiang and Wong 2008), allowing two mismatches. The target genes identified in this manner were then checked for presence of ping-pong signatures. Subsequently, ping-pong targets, as well as genes with general piRNA coverage, were compared among different species to find homologous genes, using data on gene homology extracted from Ensembl Biomart (ensembl.org/biomart, Kinsella et al. 2011). Target genes were used as input for GO-term enrichment analysis using the gene ontology web tool (geneontology.org/page/go-enrichment-analysis, Ashburner et al. 2000; The Gene Ontology Consortium 2017). Reference genes for the GO analysis were extracted from testis-expression data from the EMBL-EBI Expression Atlas database (Petryszak et al. 2016). The same data set was used for analysis of tissue-enriched genes, where enrichment was defined as at least 10-fold expression in contrast to all other tissues. The obtained results where validated by comparison with information available at the human protein atlas (proteinatlas.org, Uhlén et al. 2015).

### Analysis of Genomic Environments of piRNA Clusters

For the analysis of the genomic environment of piRNA clusters, we divided the respective genome into windows of 1 million base pairs (Mb) and used repeatmasker output and GFF gene annotation data to get the frequency for each repeat family, as well as for pseudogenes and genes per Mb. Centromeric regions, of which location information of the respective genome was obtained from the UCSC genome server, were excluded from the analysis. Furthermore, piRNA clusters were grouped by their internal gene and pseudogene content, based on GFF gene annotation. In addition, the GC content of complete genomes and of piRNA clusters was calculated using unmasked sequences, ignoring ambiguous bases. Human testis ChIP-seq data were obtained from ENCODE (encodeproject.org).

### Data Deposition

Perl and R scripts used for analyses in this study (supplementary table S5, Supplementary Material online), as well as relevant files are freely available at GitHub (github.com/d-gebert/primate-pic-evo). Small RNA transcriptome sequencing data from testis of *M.murinus* and *L.tardigradus* are deposited at NCBI’s SRA under the BioProject accession PRJNA486459 (supplementary table S4, Supplementary Material online).

## Supplementary Material

Supplementary data are available at *Genome Biology and Evolution* online.

## Supplementary Material

Supplementary Data
