# Supplementary material for: Primate piRNA Cluster Evolution Suggests Limited Relevance of Pseudogenes in piRNA-Mediated Gene Regulation
Source: Genome Biol Evol. 2019 Mar 19;11(4):1088–104. doi: 10.1093/gbe/evz060 (PMC6461890; doi:10.1093/gbe/evz060)
Supplement: Supplementary Data [file evz060_supp.pdf]

Supplementary figures

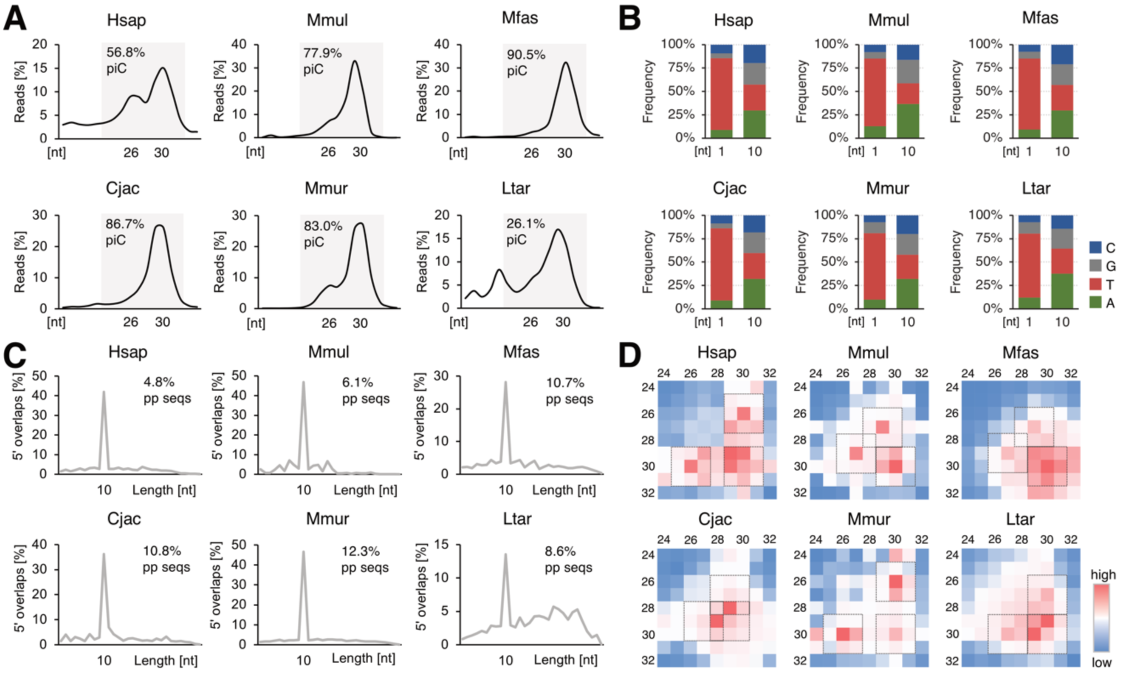

Figure S1. Basic analysis of primate testis sRNA transcriptome datasets for piRNA traits. (A) Read length distributions. Grey areas show typical mammalian piRNA size range of 24-32 nt. Percentage indicates shares of clustered 24-32 nt reads on genome, predicted by proTRAC. (B) Nucleotide frequencies in mapped reads of positions 1 and 10 starting from 5' end. (C) 5' overlaps of sense and antisense reads on genomic sequence. Percentage shows share of non-redundant sequences with ping-pong partner reads. (D) Matrices for frequencies of read length combinations in ping-pong pairs (Pairs of reads with 10 nt 5' overlaps). Dotted squares mark inferred core size range of piRNAs bound to specific PIWI proteins.

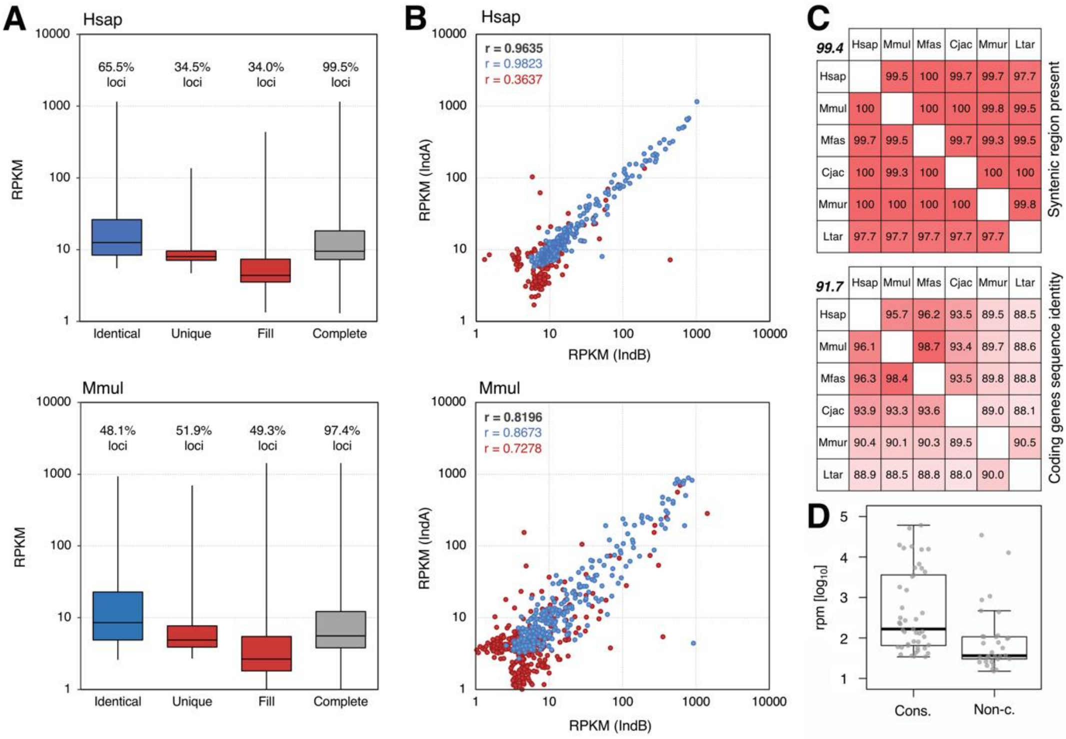

Figure S2. (A) Shares of piRNA cluster loci (piCs) predicted from samples of two individuals of the same species (*H. sapiens* and *M. mulatta*) and their read densities (RPKM; reads per kilo base per million mapped reads). Identical: loci that are found in both samples with strict prediction. Unique: loci that are found only in one the other sample with strict prediction. Fill: loci predicted with less strict threshold options that are identical to 'unique' loci in the other sample. Complete: Combination of all piC loci expressed in each individual, including complementing loci predicted with less strict thresholds. (B) Correlation of read densities (RPKM) of piCs from two individuals (IndA/B) of the same species. Blue dots correspond to 'identical' piCs, red dots correspond to 'fill' piCs of A and B. Pearson's r in boldface applies to complete sets of piCs. (C) Rates of loci for which syntenic regions could be found and rates of sequence identity between cds of homologous genes. (D) Reads (in log_{10} rpm) originating from eutherian-conserved piCs (Chirn et al. 2015) that are among the 156 primate-conserved loci compared to those that are not found in both (p<0.001, Wilcoxon-Mann-Whitney test).

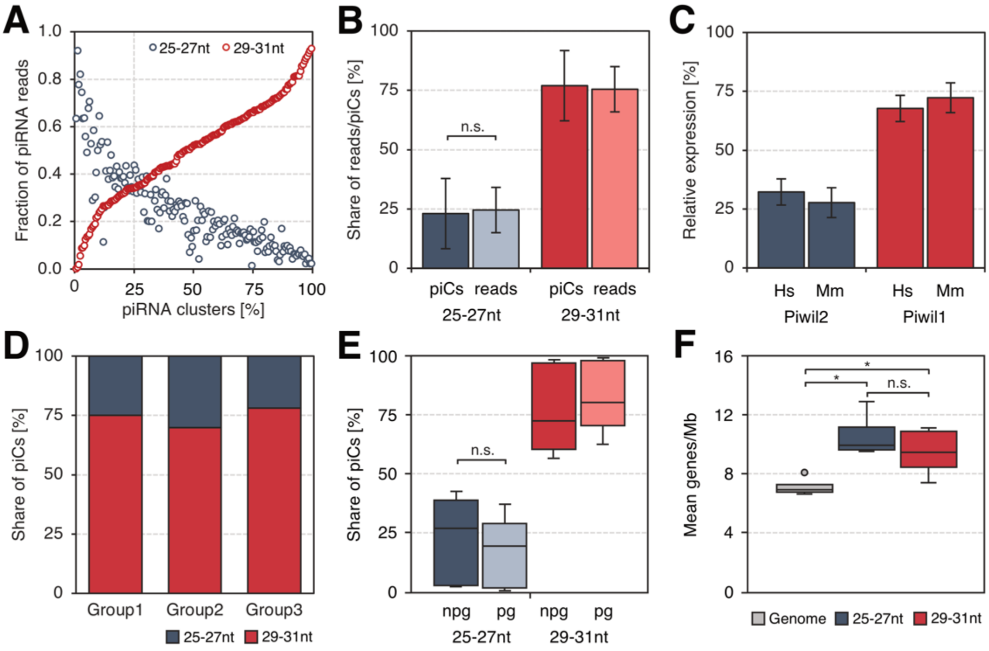

Figure S3. Analyses of piRNA clusters (piCs) divided by dominant piRNA population originating from Piwil2 (25-27nt) or Piwil1 (29-31nt). (A) Exemplary plot of read fractions with lengths of 25-27nt and 29-31nt from each piC of *L. tardigradus* sorted by 29-31nt fraction. (B) Mean shares of piCs with dominant read lengths of 25-27nt and 29-31nt (dark) and mean shares of total testis-expressed piRNA reads of the same sizes (light) of all six primates. Differences are not significant (p>0.05; paired Wilcoxon-Mann-Whitney test). (C) Mean relative mRNA expression of Piwil2 and Piwil1 in adult testis of human (Hs, n=5) and mouse (Mm, n=7). Meta analysis based on data from EMBL-EBI Expression Atlas. (D) Relative shares of primate piCs with dominant read lengths of 25-27nt and 29-31nt of loci that are present and expressed in each species (Group1), piCs that have homologs in each genome but not expressed in each species (Group2) and piCs that that do not have homologs in each genome (Group3). (E) Mean relative shares of piCs that do not contain pseudogene sequences (dark) and piCs that contain pseudogene sequences (light) divided by dominant read lengths of 25-27nt and 29-31nt. Differences are not significant (p>0.05; paired Wilcoxon-Mann-Whitney test). (F) Mean genes per Mb in genomes, and intergenic piRNA clusters with dominant read lengths of 25-27nt and 29-31nt in each primate species. *: p<0.05; n.s.: p>0.05 (paired Wilcoxon-Mann-Whitney test). Differences in genes/Mb between 25-27nt and 29-31nt piCs are not significant for each primate species (p>0.05; Wilcoxon-Mann-Whitney test).

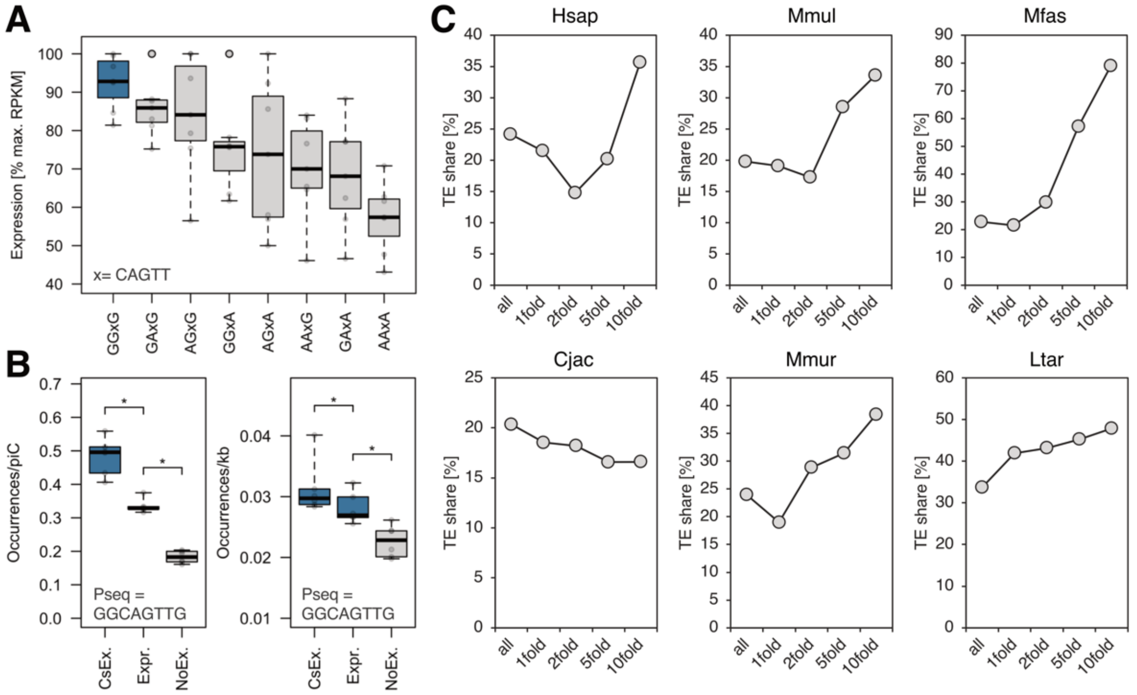

Figure S4. (A) Association of A-MYB promoter sequence variants with expression strength as relative mean read density [RPKM] of piRNA clusters (piCs) in each species. (B) Occurrences of A-MYB promoter sequence 'GGCAGTTG' per piC and per kb in expressed and non-expressed homologous piC loci in each species. *: p<0.05 (paired Wilcoxon-Mann-Whitney test); CsEx.: Conserved expression (across all species); Expr.: Expressed; NoEx.: Non-expressed; Pseq: Promoter sequence. (C) Shares of transposon sequences in piRNAs produced by highly expressed piCs (1-10fold) compared to the remaining piRNA transcriptome (all).

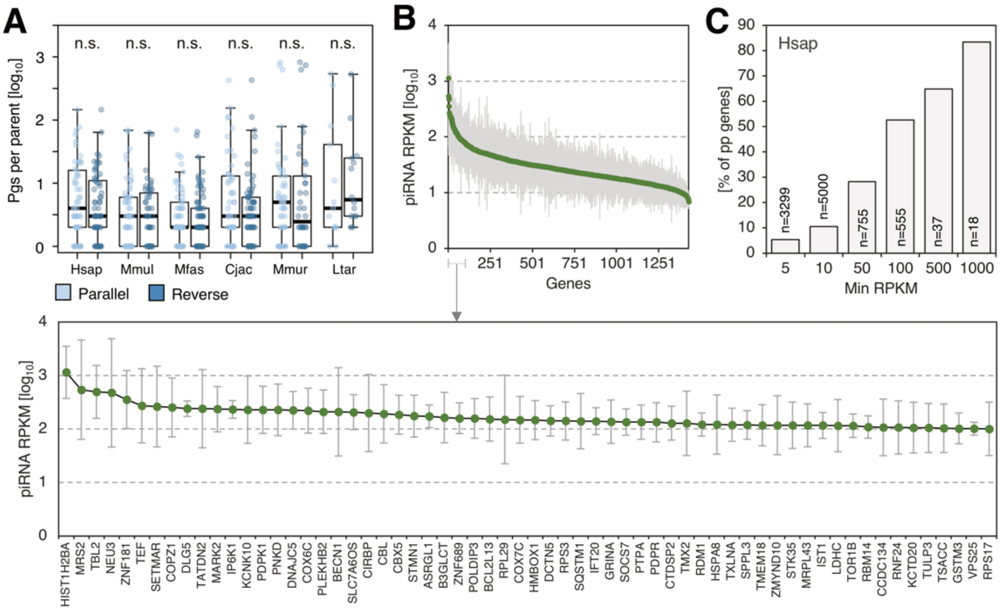

Figure S5. (A) Number of pseudogenes per parent gene for pseudogenes in piRNA clusters (piCs); n.s.: p>0.05 (Wilcoxon-Mann-Whitney test). (B) Homologous genes with piRNA coverage of at least 5 RPKM (reads per kilo base per million reads) in all six primate species ($\log_{10}$ RPKM means and standard deviations). Bottom: Genes with mean >2 $\log_{10}$ RPKM. (C) Relationship of RPKM range (e.g. 5: RPKM ≥5, <10) and share of ping-pong genes in human.

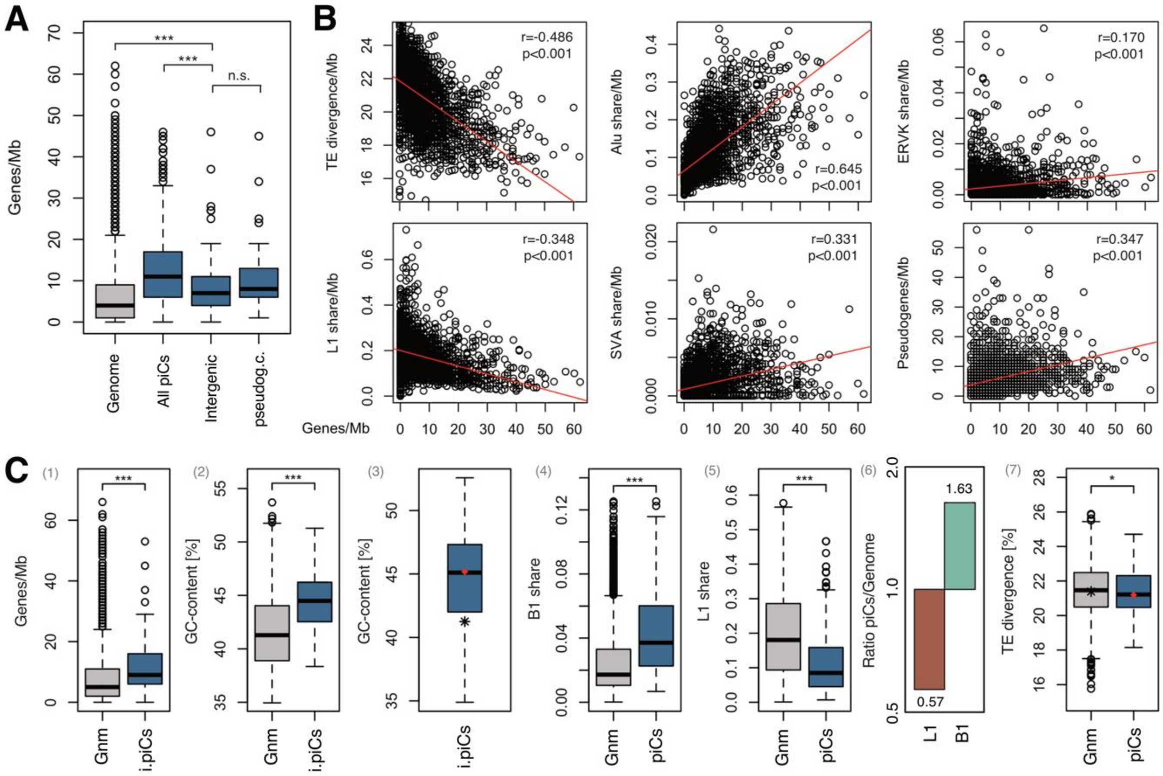

Figure S6. Genomic environments of piRNA clusters (piCs) and correlations with gene density in human and mouse. (A) Gene densities of 1 Mb genomic slices that contain piCs compared to the whole genome in human. All piCs: All human piCs; Intergenic: piCs that do not contain coding genes or pseudogenes. pseudog.c.: piCs that contain pseudogene sequence; ***: p<0.001; n.s.: p>0.05 (Wilcoxon-Mann-Whitney test). (B) Correlations of transposon divergence, shares of transposon families (Alu, L1, SVA) and pseudogene abundance with gene density (genes/Mb) in the human genome. (C) Plots 1,2,4,5,7: Gene density, GC content, B1/L1 shares and TE divergence of 1 Mb genomic slices that contain piCs compared to the whole genome in mouse. Plot 3: GC content of each piC. Plot 6: Ratios of B1/L1 sequence shares between piCs and genomic sequence. sRNA dataset: SRR772055; total RNA size-selected, testis, 6 weeks old, C57BL/6J. ***: p<0.001; *: p<0.05 (p=0.047; Wilcoxon-Mann-Whitney test). Total means of all intergenic piC sequences per species are indicated by red diamond shaped points. Means of whole genomes are shown by star shaped points (see Figure 5).

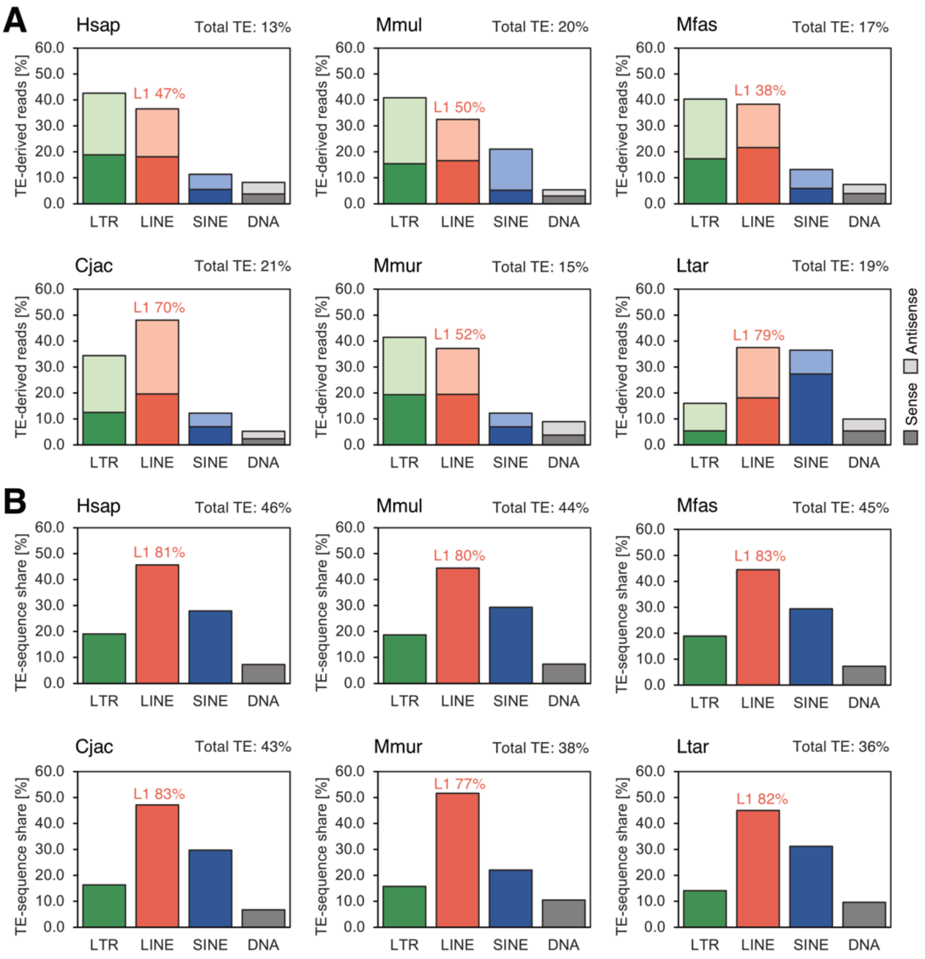

Figure S7. (A) Relative shares of transposon sequences in piRNA reads. Total TE: Fraction of all mapped piRNA reads that correspond to transposable elements. L1 %: Percentage of LINE1 reads among all LINE-associated reads. LTR: Long terminal repeat retrotransposons; LINE: Long interspersed elements; SINE: Short interspersed elements; DNA: DNA transposons. (B) Relative shares of transposon sequences in whole genomes. Total TE: Fraction TE-derived genomic sequence. L1 %: Percentage of LINE1 sequence among all LINE-associated sequence.

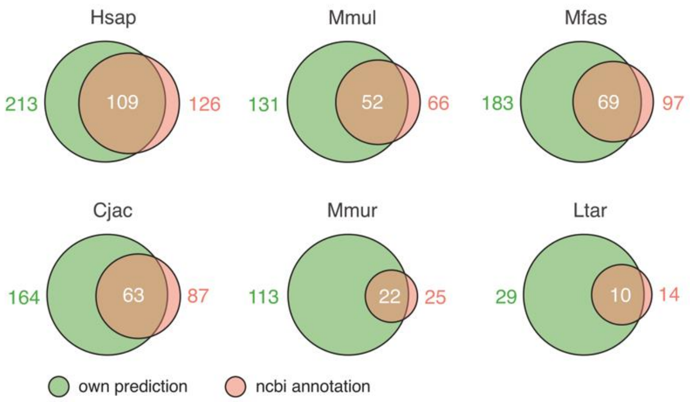

Figure S8. Comparison of NCBI annotation and custom pseudogene prediction in piRNA cluster sequences. Counts of NCBI annotated and predicted pseudogenes with number of identical pseudogenes.

Supplementary tables

PANTHER GO-Slim Biological Process	REFLIST (18235)	Genes (1459)	Genes (expected)	Genes (fold Enrichment)	Genes (raw P-value)	Genes (FDR)
oxidative phosphorylation (GO:0006119)	45	12	3.6	3.33	8.10E-04	5.20E-03
mRNA 3'-end processing (GO:0031124)	34	9	2.72	3.31	3.71E-03	1.71E-02
protein folding (GO:0006457)	85	22	6.8	3.23	9.96E-06	1.16E-04
protein methylation (GO:0006479)	49	12	3.92	3.06	1.52E-03	8.81E-03
spermatogenesis (GO:0007283)	59	14	4.72	2.97	8.29E-04	5.19E-03
tRNA metabolic process (GO:0006399)	111	22	8.88	2.48	3.37E-04	2.41E-03
RNA catabolic process (GO:0006401)	66	13	5.28	2.46	7.40E-03	2.95E-02
respiratory electron transport chain (GO:0022904)	102	19	8.16	2.33	2.38E-03	1.21E-02
rRNA metabolic process (GO:0016072)	107	19	8.56	2.22	2.98E-03	1.43E-02
mRNA processing (GO:0006397)	238	42	19	2.21	1.43E-05	1.51E-04
translation (GO:0006412)	193	34	15.44	2.2	9.42E-05	7.93E-04
protein targeting (GO:0006605)	167	29	13.36	2.17	3.09E-04	2.36E-03
mRNA splicing, via spliceosome (GO:0000398)	173	28	13.84	2.02	1.06E-03	6.48E-03
RNA splicing, via transesterification reactions (GO:0000375)	151	23	12.08	1.9	7.90E-03	3.11E-02
generation of precursor metabolites and energy (GO:0006091)	166	24	13.28	1.81	1.12E-02	4.14E-02
protein metabolic process (GO:0019538)	1434	198	114.74	1.73	1.59E-12	7.77E-11
chromatin organization (GO:0006325)	254	35	20.32	1.72	4.31E-03	1.88E-02
proteolysis (GO:0006508)	428	54	34.24	1.58	2.60E-03	1.27E-02
protein localization (GO:0008104)	503	63	40.25	1.6	1.09E-03	6.48E-03
RNA metabolic process (GO:0016070)	1488	176	119.06	1.48	9.10E-07	1.31E-05
intracellular protein transport (GO:0006886)	670	78	53.61	1.46	2.10E-03	1.13E-02
organelle organization (GO:0006996)	1149	132	91.93	1.44	9.00E-05	7.54E-04
cellular protein modification process (GO:0006464)	759	87	60.73	1.43	1.95E-03	1.08E-02
cellular component biogenesis (GO:0044085)	730	83	58.41	1.42	3.20E-03	1.49E-02
response to stress (GO:0006950)	528	60	42.25	1.42	1.29E-02	4.55E-02
catabolic process (GO:0009056)	1117	126	89.37	1.41	2.70E-04	2.11E-03
protein transport (GO:0015031)	709	79	56.73	1.39	5.46E-03	2.26E-02
primary metabolic process (GO:0044238)	4428	484	354.29	1.37	2.48E-13	1.51E-11
nucleobase-containing compound metabolic process (GO:0006139)	2644	287	211.55	1.36	2.82E-07	4.60E-06
biosynthetic process (GO:0009058)	1677	177	134.18	1.32	3.30E-04	2.44E-03
metabolic process (GO:0008152)	5502	579	440.22	1.32	1.24E-13	1.01E-11
cellular component organization or biogenesis (GO:0071840)	1924	199	153.94	1.29	3.74E-04	2.61E-03
cellular component organization (GO:0016043)	1795	179	143.62	1.25	3.70E-03	1.70E-02
nitrogen compound metabolic process (GO:0006807)	2404	231	192.35	1.2	5.12E-03	2.19E-02
regulation of biological process (GO:0050789)	1974	124	157.94	0.79	5.42E-03	2.28E-02

Table S1. GO term analysis: Panther GO slim biological process. Test: Fisher's Exact with FDR (false discovery rate) multiple test correction. Genes: 1428 orthologous genes with piRNA coverage in all six species. RefList: Testis-expressed genes.

PANTHER GO-Slim Molecular Function	REFLIST (18235)	Genes (1459)	Genes (expected)	Genes (fold Enrichment)	Genes (raw P-value)	Genes (FDR)
translation regulator activity (GO:0045182)	77	17	6.2	2.76	4.95E-04	1.18E-02
mRNA binding (GO:0003729)	134	27	10.72	2.52	5.57E-05	1.77E-03
structural constituent of ribosome (GO:0003735)	121	23	9.68	2.38	5.61E-04	1.07E-02
RNA binding (GO:0003723)	370	58	29.6	1.96	8.81E-06	3.35E-04
catalytic activity (GO:0003824)	4006	377	320.52	1.18	7.51E-04	1.30E-02

Table S2. GO term analysis: Panther GO slim molecular function. Test: Fisher's Exact with FDR (false discovery rate) multiple test correction. Genes: 1428 orthologous genes with piRNA coverage in all six species. RefList: Testis-expressed genes.

PANTHER GO-Slim Cellular Component	REFLIST (18235)	Genes (1459)	Genes (expected)	Genes (fold Enrichment)	Genes (raw P-value)	Genes (FDR)
mitochondrial inner membrane (GO:0005743)	107	21	8.6	2.45	5.10E-04	2.04E-03
ribosome (GO:0005840)	154	28	12.32	2.27	2.72E-04	1.16E-03
ribonucleoprotein complex (GO:0030529)	413	67	33.04	2.03	5.34E-07	3.42E-06
nuclear outer membrane-endoplasmic reticulum membrane network (GO:0042175)	228	33	18.24	1.81	2.65E-03	8.92E-03
cytosol (GO:0005829)	496	70	39.69	1.76	2.05E-05	1.01E-04
endoplasmic reticulum (GO:0005783)	400	56	32	1.75	1.86E-04	8.49E-04
mitochondrion (GO:0005739)	382	51	30.56	1.67	1.10E-03	4.03E-03
macromolecular complex (GO:0032991)	1977	248	158.18	1.57	1.56E-11	1.66E-10
cytoplasm (GO:0005737)	3037	370	242.99	1.52	7.35E-16	2.35E-14
nucleoplasm (GO:0005654)	379	45	30.3	1.48	1.45E-02	4.03E-02
protein complex (GO:0043234)	1630	192	130.42	1.47	3.57E-07	2.50E-06
nucleus (GO:0005634)	1857	214	148.58	1.44	3.10E-07	2.47E-06
organelle (GO:0043226)	3718	416	297.48	1.4	1.50E-12	1.93E-11
intracellular (GO:0005622)	5019	556	401.6	1.38	4.48E-17	2.87E-15
cell part (GO:0044464)	5256	565	420.54	1.34	7.30E-15	1.56E-13

Table S3. GO term analysis: Panther GO slim cellular component. Test: Fisher's Exact with FDR (false discovery rate) multiple test correction. Genes: 1428 orthologous genes with piRNA coverage in all six species. RefList: Testis-expressed genes.

BioSample id	SRA id	Species	Sex	Tissue	Developmental Stage / Age
SAMN02029047	SRR835325	Homo sapiens	Male	Testis	adult (34 years)
SAMN02029046	SRR835324	Homo sapiens	Male	Testis	adult (59 years)
SAMN00216771	SRR116839	Macaca mulatta	Male	Testis	adult (9 years)
SAMN01161964	SRR553581	Macaca mulatta	Male	Testis	adult (8 years)
SAMN03283413	SRR1755243	Macaca fascicularis	Male	Testis	adult (7 years)
SAMN02429366	SRR1041905	Callithrix jacchus	Male	Testis	adult (3 years)
SAMN09847006	SRR7704801	Microcebus murinus	Male	Testis	adult (>1 year)
SAMN09847007	SRR7704800	Loris tardigradus	Male	Testis	adult (>1 year)

Table S4. Tissue sample information.

Script	Description	Method section
get_pp_partners.pl	Count reads with ping-pong partners and make matrix of read counts for each partner length combination	Basic analyses
merge_pic_loci.pl	Merge piRNA cluster loci with a distance less than 10 kb	piRNA cluster prediction
compare_strict_pics.pl	Compare piRNA clusters predicted with strict settings within the same species	piRNA cluster prediction
compare_all_pics.pl	Compare piRNA clusters predicted with strict settings within the same species, including less strictly predicted loci	piRNA cluster prediction
find_pic_hom_loci.pl	Identify homologous piRNA clusters in another species by finding syntenic regions and searching for sequence similarity with blastn/dc-megablast	Homologous piRNA cluster identification
get_hom_loc_lineages.pl	Combine pairs of homologous piRNA cluster loci to get lineages of homologous loci across several species	Homologous piRNA cluster identification
get_hom_loc_identities.pl	Extract information on rates of identified syntenic regions, found homologous loci, expressed piRNA clusters and sequence similarities of homologous loci	Homologous piRNA cluster analyses
blast_genomes.pl	Call dc-megablast on all combinations of given (repeatmasked) genome files	Homologous piRNA cluster analyses
blast_cds.pl	Call dc-megablast on all combinations of given cds files	Homologous piRNA cluster analyses
get_genome_identities.pl	Extract information on sequence similarities between genomes	Homologous piRNA cluster analyses
get_cds_identities.pl	Extract information on sequence similarities between cds of genes	Homologous piRNA cluster analyses
extract_all_pic_loci.pl	Extract information on all identified piRNA clusters	Homologous piRNA cluster analyses
ids_to_hom_loc_lineages.pl	Add piRNA cluster ids assigned by proTRAC to lineages of homologous loci and extract cluster expression rates	Homologous piRNA cluster analyses
get_hom_loc_TE_divs.pl	Get TE divergences for piRNA cluster regions from repeatmasker files	Homologous piRNA cluster analyses
heatmapper.R	Create expression heatmap with dendrogram for homologous piRNA clusters using hierarchical clustering, average linkage and pearson distance	Homologous piRNA cluster analyses
predict_pseudogenes.pl	Search for sequences similar to cds using dc-megablast in regions not annotated as genes and combine close hits to predict pseudogenes	Pseudogene prediction
get_pic_pseudogenes.pl	Extract pseudogenes in piRNA cluster regions from genome-wide annotation	Pseudogene analyses
compare_predictions.pl	Compare pseudogene prediction and NCBI annotation within cluster loci	Pseudogene analyses
get_pseudogene_info.pl	Extract information on orientation, type and parents of pseudogenes in the whole genome and within cluster loci	Pseudogene analyses
find_pic_hom_pseudogenes.pl	Search for homologous pseudogene sequences in homologous piRNA cluster loci across species with dc-megablast	Pseudogene analyses
get_pir_target_genes.pl	Use output file generated by seqmap, mapping piRNA reads on cdna, to find general targets and ping-pong genes	Target gene analyses
get_pic_target_genes.pl	Map antisense piRNA reads produced from pseudogenes in piRNA clusters on cdna using seqmap to find general targets and ping-pong genes	Target gene analyses
get_pir_target_orthologs.pl	Find orthologous genes targeted by piRNAs in general	Target gene analyses
get_pic_target_orthologs.pl	Find orthologous genes targeted by pseudogene-derived piRNAs	Target gene analyses
get_pir_target_3utrs.pl	Get 3'-UTR lengths and compare to piRNA coverage	Target gene analyses
get_pic_environments.pl	Scan whole genome with 1 Mb window and get information on TE shares, gene density, pseudogene density, GC content and piRNA cluster locations	Analysis of genomic environments
get_gc_share.pl	Calculate total GC share of genome/piRNA clusters	Analysis of genomic environments

Table S5. Main Perl and R scripts developed and used in this study (github.com/d-gebert/primate-pic-evo).

Supplementary Methods

Comparison of piRNA clusters among individuals of the same species

Since proTRAC most critically relies on read density and locus size, the applied thresholds inevitably lead to sharp cutoffs in the long tails of the distributions, which might have the effect that the comparability between different samples can be problematic. Indeed, if piRNA clusters are predicted in a stringent manner from samples of two individuals from the same species, e. g. *H. sapiens*, only 65.5% of loci are identical, while 34.5% seem to be unique to the respective individual (Figure S2). However, using piRNA clusters predicted with less strict options to find loci that are identical to those more strictly predicted that appear to be unique, overall 99.5% of piRNA clusters were found to be expressed in both individuals. Similarly, when comparing two specimen of *M. mulatta*, only 48.1% of loci overlap, which can be increased to 97.4% in the same manner. As expected, the seemingly unique loci are predominantly shifted towards the lower end of the read density (RPKM; reads per kilo base per million mapped reads) spectrum relative to those that have identical equivalents in each individual, while loci that were predicted with less strict options to fill the missing counterparts fall mostly below that range (Figure S2). Apart from read density, thresholds for cluster size and minimum fraction of reads with 1T (1U) or 10A have a similar, though less marked effect for the prediction of piRNA clusters with the respective properties that come close to these thresholds.

Pseudogene prediction

To verify the validity of our custom pseudogene annotation method, we compared our results for piRNA cluster regions to GFF gene annotation data from NCBI (Figure S8). We could predict on average 2.4 times the number of annotated pseudogenes, including 78% of annotated sequences, showing the effectiveness of our approach.
